# High apolipoprotein M serum levels correlate with chronic obstructive pulmonary disease

**DOI:** 10.1186/s12944-016-0228-1

**Published:** 2016-03-22

**Authors:** Hui Li, Yinyin Liu, Ling Wang, Ting Shen, Wenhan Du, Zhijun Liu, Ruohong Chen, Min Hu

**Affiliations:** Department of Clinical Laboratory, The Second Xiangya Hospital, Centre South University, Changsha, 410011 Hunan Province China; Department of Pulmonary Medicine, The Second Xiangya Hospital, Centre South University, Changsha, 410011 Hunan Province China

**Keywords:** Apolipoprotein M, Chronic obstructive pulmonary disease, Forced expiratory volume in one second, % predicted FEV_1_, Cardiovascular disease

## Abstract

**Background:**

Recently, variations in a component of high-density lipoprotein (HDL), namely apolipoprotein M (apoM), were found to be associated with chronic obstructive pulmonary disease (COPD). The aim of this study was to evaluate the association between apoM and COPD severity. Factors associated with apoM, COPD, or coronary artery disease (CAD) were also assessed.

**Methods:**

A total of 110 COPD patients and 110 age- and sex-matched non-COPD controls were included. Among them, thirty COPD patients and seven non-COPD controls had CAD. ApoM and pentraxin-3 levels were measured by ELISA. Additionally, the levels of high-sensitivity C-reactive protein (hs-CRP), cholesterol, and triglyceride were assessed using an automatic biochemical analyzer.

**Results:**

Serum apoM levels increased gradually with COPD severity, with the most prominent apoM elevation observed in very severe COPD cases. In addition, ApoM was correlated with percent-predicted forced expiratory volume in one second (% predicted FEV1) (*r* = −0.38, *P* < 0.001), low-density lipoprotein cholesterol (LDL-C) (*r* = 0.23, *P* < 0.017), and hs-CRP (*r* = 0.24, *P* = 0.01) in COPD patients. Furthermore, apoM was shown to be a risk factor for COPD onset (OR = 1.095, 95 % CI = 1.034–1.160, *P* = 0.002), but not associated with CAD in COPD patients.

**Conclusions:**

Serum apoM was elevated in COPD patients and increased gradually with COPD severity. However, there was no association between apoM and CAD development in COPD patients.

**Electronic supplementary material:**

The online version of this article (doi:10.1186/s12944-016-0228-1) contains supplementary material, which is available to authorized users.

## Background

Chronic obstructive pulmonary disease (COPD), the fourth leading cause of morbidity and mortality worldwide, is associated with significant economic and social burden [1]. An increasing number of COPD patients die from systemic inflammatory complications, such as cardiovascular diseases, rather than respiratory system failure [2, 3]. However, the mechanisms linking systemic inflammation to cardiovascular diseases remain unclear and cannot simply be explained by the effects of smoking or hypertension [3].

Apolipoprotein M (apoM) is a 26-kDa apolipoprotein that belongs to the lipocalin protein superfamily. In the serum, apoM mainly binds to high-density lipoproteins (HDL). ApoM-HDL interactions promote pre-β HDL formation [4] and increase cholesterol efflux from foam cells [5]. Moreover, the anti-atherosclerotic properties of apoM are attributed to its anti-inflammatory activity [6] and protect low-density lipoprotein cholesterol (LDL-C) against oxidation [7]. ApoM can potentially protect the endothelium under homeostatic conditions [8] and suppress lymphopoiesis and neuroinflammation during the adaptive immune response [9, 10].

The human apoM gene is located in the major histocompatibility complex class III region of chromosome 6, which also contains several genes involved in immune and inflammatory responses [11]. Recent studies suggested that a number of inflammatory factors might be correlated with increased serum apoM levels in COPD patients [12, 13]. Interestingly, serum and lung platelet-activating factor and leptin levels are increased in COPD patients and correlated with disease severity [14, 15], suggesting that the systemic inflammation associated with COPD might cause an increase in apoM expression. As part of the MESA COPD study, Burkart et al. [16] recently reported that two single nucleotide polymorphisms flanking the apoM gene are associated with altered lung function. The latter study also revealed that apoM mRNA is inversely associated with the FEV_1_/FVC ratio [16].

The associations between apoM expression and markers of inflammation remain unknown in COPD patients. We hypothesized that serum apoM levels correlate with the onset and severity of COPD. The main purpose of this study was to assess the serum apoM level and determine its clinical associations with lung function and inflammatory markers in a cohort of patients with COPD at different stages of disease severity.

## Methods

### Subjects

This was a case–control study of 110 COPD patients evaluated at The Second Xiangya Hospital between July 2014 and June 2015. The diagnosis of COPD was based on each patient’s clinical history, physical examination, chest radiography, arterial blood gas measurements, and lung function, according to the Global Initiative for Chronic Obstructive Lung Disease (GOLD) 2015 classification [17]. Patients in this study were classified as GOLD stages I/II (*n* = 32), III (*n* = 39), and IV (*n* = 39).

The inclusion criterion was COPD patients with an FEV_1_/FVC < 0.7. Exclusion criteria were: 1) body mass index (BMI) >3 0 kg/m^2^; 2) history of pulmonary embolism; 3) metabolic acidosis; 4) immunological disease; 5) cancer; 6) venous or arterial thrombosis; 7) renal disease.

Coronary artery disease (CAD) was diagnosed by the presence of stenosis on coronary angiography. Hypertension was defined as systolic blood pressure ≥ 140 mmHg and/or diastolic blood pressure ≥ 90 mmHg. Diabetes mellitus was defined as fasting plasma glucose ≥ 7.0 mmol/L and 2-h glucose tolerance test glucose > 11.1 mmol/L.

The control group included 110 age and sex matched non-COPD volunteers who underwent physical examination in our hospital, with FEV_1_/FVC < 0.7 and a normal chest X-ray.

The study protocol was reviewed and approved by The Second Xiangya Hospital Investigational Review Board. Written informed consent was obtained from all participants.

### Blood sampling

After an overnight fast and at least 20 min of rest, blood samples were collected from each subject. Serum was obtained by centrifugation at 3500 rpm for 5 min, and aliquots were stored at −80 °C.

### Enzyme-linked immunosorbent assay (ELISA) for apoM assessment

Serum apoM levels were measured using a sandwich ELISA (Yuan Tai Bio Inc., Changsha, Hunan, People’s Republic of China). Optical density (OD) was measured at 450 nm (with a background reading at 620 nm) on an ELX-800 absorbance reader (BioTek Instruments, Inc., Winooski, VT, USA). The concentration of apoM (as mg/L) in each sample was derived from a standard curve.

### Lipoprotein and inflammation marker levels

Serum triglyceride, total cholesterol, HDL cholesterol (HDL-C), LDL-C, apolipoprotein A (apoA), apolipoprotein B (apoB), lipoprotein(a), and high-sensitivity C-reactive protein (hs-CRP) levels were measured on an ARCHITECT c8000 System (Abbott Laboratories, Irving, TX, USA). Pentraxin-3 levels were detected using an ELISA kit (CUSABIO, Wuhan, China) according to the manufacturer’s instructions.

### Statistical analysis

Continuous data are presented as the mean ± standard deviation; categorical data are expressed as percentages. Continuous data were analyzed by Student’s *t*-test or one-way analysis of variance (ANOVA) with Tukey’s test, as appropriate. Variables with a skewed distribution were converted by logarithmic transformation before analysis. Pearson’s correlation analysis was used to assess the associations between the apoM level and normally distributed variables. Spearman correlation analysis was used for variables with skewed distributions. Binary logistic regression models were carried out to identify the risk factors for COPD onset and identify the risk factors for CAD in COPD patients. All analyses were performed with SPSS 20.0 (SPSS, USA) or GraphPad Prism 5.0 (GraphPad Software, La Jolla, CA, USA). Two-sided *P* < 0.05 was considered statistically significant.

## Results

### Patient characteristics

**Fig. 1:**
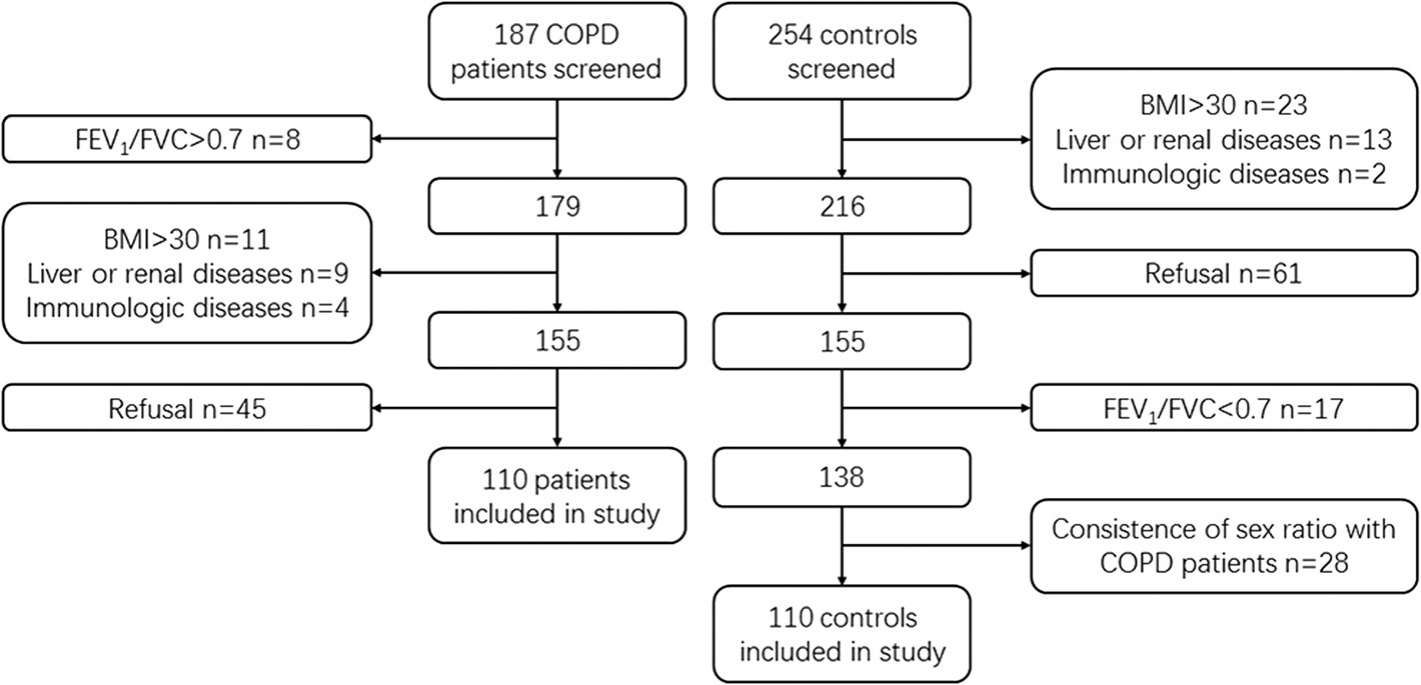
Patient flowchart

Figure 1 depicts the patient flowchart. From 187 COPD patients screened for participation, 32 did not meet the eligibility criteria and 45 refused to participate; therefore, 110 patients were finally assessed. Additionally, 254 controls were screened for participation; 45 failed the eligibility criteria, 61 refused to participate, and 28 were eliminated by the matching criteria; therefore, 110 were finally included.

Age (67 ± 10 vs. 67 ± 10 years, *P* = 0.823), gender (86 % vs. 86 % men, *P* = 0.99), and BMI (22.2 ± 2.3 vs. 22.2 ± 2.3 kg/m^2^, *P* = 0.954) were similar between the COPD and control groups (Table 1) and across all COPD GOLD stages (Additional file 1: Table S1). The COPD group had a higher proportion of smokers (64 % vs. 35 %, *P* < 0.001), CAD (28 % vs. 6 %, *P* < 0.001), hypertension (25 % vs. 11 %, *P* = 0.008), and hormone therapy (27 % vs. 0 %, *P* < 0.001) compared with the control group. % predicted FEV_1_ and FEV_1_/FVC were lower in COPD patients compared with control values (*P* < 0.001). Pentraxin-3 levels were higher in COPD patients compared with the control group (1.28 ± 2.66 vs. 0.96 ± 2.05 ng/L, *P* = 0.002).

**Table 1:** Demographic and biochemical parameters of the study subjects

	COPD	Controls	*P* value
Subjects, n	110	110	
Demographics			
Age, years	67 ± 10	67 ± 10	0.823
Sex, % male	86	86	0.847
Body mass index, kg/m^2^	22.2 ± 2.3	22.2 ± 2.3	0.954
Smoking history, %	64	35	<0.001
GOLD stages, n			
GOLD I/II	32		
GOLD III	39		
GOLD IV	39		
Comorbidities, %			
CAD	28	6	<0.001
Hypertension	25	11	0.008
Diabetes mellitus	5	1	0.098
Treatment, %			
Prednisone	27	0	<0.001
Aminophylline	23	0	<0.001
β-2 adrenergic receptor antagonist	22	7	0.017
Anticholinergics	23	0	<0.001
Lung function			
FEV_1_% predicted	43.81 ± 19.77	92.34 ± 8.29	<0.001
FEV_1_/FVC	49.44 ± 15.96	97.48 ± 3.73	<0.001
Lipids profile			
Triglycerides, mmol/L	1.17 ± 0.67	1.43 ± 1.11	0.036
Total cholesterol, mmol/L	4.16 ± 0.82	4.49 ± 0.76	0.003
HDL-C, mmol/L	1.15 ± 0.30	1.31 ± 0.26	<0.001
LDL-C, mmol/L	2.50 ± 0.77	2.61 ± 0.70	0.285
Apolipoprotein A, g/L	1.36 ± 0.29	1.46 ± 0.27	0.011
Apolipoprotein B, g/L	0.89 ± 0.22	0.94 ± 0.27	0.122
Apolipoprotein M, mg/L	23.08 ± 9.54	17.02 ± 4.74	<0.001
Lipoprotein(a), g/L	163.9 ± 2.4	99.0 ± 9.3	<0.001
Inflammatory factors			
Hs-CRP, mg/L	11.52 ± 3.97	1.47 ± 2.77	<0.001
Pentraxin-3, ng/L	1.28 ± 2.66	0.96 ± 2.05	0.002

Data are mean ± standard deviation. Values of triglycerides, lipoprotein(a), and hs-CRP were converted into a logarithmic form before analysis. The *p*-values refer to comparisons between the COPD and control groups

*GOLD* Global Initiative for Chronic Obstructive Lung Disease, *COPD* chronic obstructive pulmonary disease, *CAD* coronary artery disease, *HDL-C* high-density lipoprotein cholesterol, *LDL-C* low-density lipoprotein cholesterol, *hs-CRP* high-sensitivity C-reactive protein, *FEV*_*1*_ forced expiratory volume in one second, *FVC* forced vital capacity, *NS* non-significant

### Serum apoM amounts are elevated in COPD patients

**Fig. 2:**
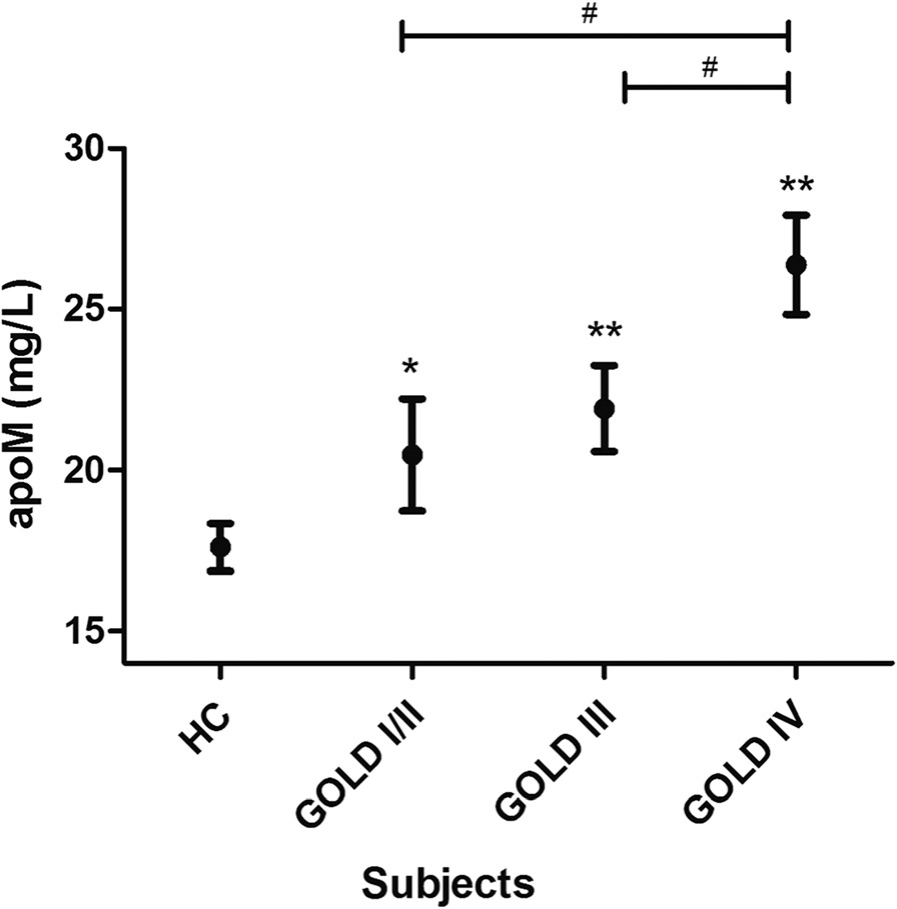
Serum apolipoprotein M (apoM) is elevated in chronic obstructive pulmonary disease (COPD) patients. Serum concentrations of apoM in COPD patients, grouped according to the Global Initiative for Chronic Obstructive Lung Disease (GOLD) stage, and healthy control subjects (HC). Error bars represent the standard errors of the mean. *P < 0.05, **P < 0.01 vs. HC. ^#^ *P* < 0.05, between two groups indicated by the horizontal line

Serum apoM levels in COPD patients were 23.08 ± 9.54 mg/L, higher than values obtained for healthy controls (*P* < 0.0001; Table 1). The most pronounced apoM increase in COPD patient subpopulations was observed in GOLD stage IV, followed by stages III and I/II (*P* < 0.05; Fig. 2).

### Factors associated with apoM in COPD patients

**Fig. 3:**
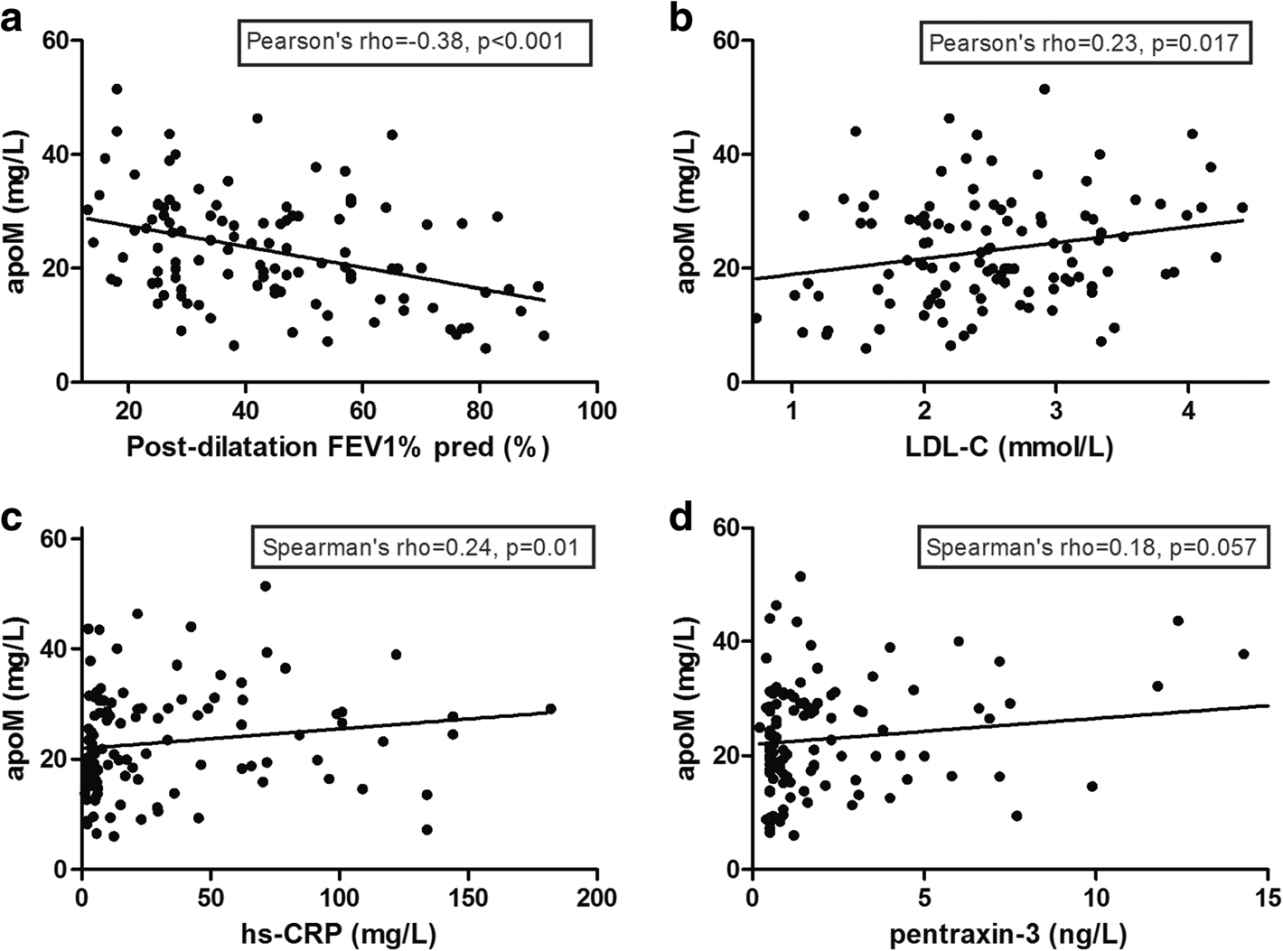
Associations of apoM and % predicted FEV_1_, LDL-C, hs-CRP, and Pentraxin-3. **a** Post-dilatation forced expiratory volume in one second, % predicted FEV_1_, was correlated with apoM (r = −0.38, P < 0.001); and (**b**) low-density lipoprotein cholesterol (LDL-C) was correlated with apoM (r =0.23, P = 0.017). **c** High-sensitivity C-reactive protein (hs-CRP) was associated with apoM (*r* = 0.24, *P* = 0.01). **d** Pentraxin-3 was not associated with apoM (*r* = 0.18, *P* = 0.057)

The serum lipid profile and pulmonary function were assessed for their associations with apoM using univariate correlation analyses; % predicted FEV_1_ was used to evaluate lung function. Interestingly, % predicted FEV_1_ was inversely correlated with the serum apoM level (*r* = −0.38, *P* < 0.001; Fig. 3a). ApoM was positively correlated with serum LDL-C (*r* = 0.23, *P* = 0.017; Fig. 3b) and hs-CRP (*r* = 0.24, *P* = 0.01; Fig. 3c). Marginally significant correlations were observed between apoM and pentraxin-3 (*r* = 0.18, *P* = 0.06; Fig. 3d).

### Risk factors for COPD onset

To identify the risk factors associated with the onset of COPD symptoms, a binary logistic regression model of the whole study population was built by initially including all variables with *P* < 0.05 in univariate analyses (Table 1) and systematically excluding variables that were not significant (*P* > 0.10). Triglycerides (OR = 0.528, 95 % CI = 0.283–0.985, *P* = 0.045) and HDL-C (OR = 0.292, 95 % CI = 0.101-0.844, *P* = 0.023) were identified as protective factors. In contrast, apoM (OR = 1.095, 95 % CI = 1.034–1.160, *P* = 0.002), smoking history (OR = 2.542, 95 % CI = 1.188–5.440, *P* = 0.016), and hs-CRP (OR = 1.249, 95 % CI = 1.136–1.374, *P* < 0.001) were found to be risk factors for COPD onset in the final model (Table 2).

**Table 2:** Binary logistic regression analysis for COPD

	*P* value	OR	95 % CI (OR)
Hs-CRP	<0.001	1.249	1.136–1.374
Smoking history	0.016	2.542	1.188–5.440
Triglycerides	0.045	0.528	0.283–0.985
HDL-C	0.023	0.292	0.101–0.844
Apolipoprotein A	0.063	5.830	0.911–37.293
Apolipoprotein M	0.002	1.095	1.034–1.160

Note: High-density lipoprotein cholesterol was transformed to a dichotomous variable according to its average value 1.24 mmol/L

*COPD* chronic obstructive pulmonary disease, *hs-CRP* high-sensitivity C-reactive protein, *HDL-C* high-density lipoprotein cholesterol

### Risk factors for CAD in COPD patients

To identify the risk factors associated with CAD in COPD patients, a binary logistic regression model was performed. Non-significant variables (*P* > 0.05) were systematically excluded. Use of a β-2 adrenergic receptor antagonist (OR = 0.179, 95 % CI = 0.040–0.804, *P* = 0.025) was identified as a protective factor. In contrast, hypertension (OR = 2.018, 95 % CI = 1.380-2.952, *P* < 0.001) and aminophylline use (OR = 7.048, 95 % CI = 2.169-22.906, *P* = 0.001) were risk factors for CAD onset in COPD patients (Table 3). In this model, apoM was not associated with the development of CAD in COPD patients.

**Table 3:** Binary logistic regression analysis for CAD in COPD patients

	*P*	OR	95 % CI (OR)
Hypertension	<0.001	2.018	1.380–2.952
Aminophylline use	0.001	7.048	2.169–22.906
β-2 adrenergic receptor antagonist use	0.025	0.179	0.040–0.804

*COPD* chronic obstructive pulmonary disease, *CAD* coronary artery disease

## Discussion

This study revealed a relationship between the serum apoM level and % predicted FEV_1_, a gold standard for severity assessment of airflow obstruction. These results extend the findings of the MESA COPD study, which demonstrated an inverse relationship between airflow obstruction and apoM mRNA levels and suggested that serum apoM is a predictor of COPD onset [16]. As shown above, serum apoM was not associated with CAD in COPD patients. A potential mechanism behind the high cardiovascular disease prevalence in COPD patients is the altered endothelium function resulting from airflow obstruction [18]. Previous studies demonstrated that the type or degree of endothelial damage in COPD patients might be involved in the development of CAD [19, 20], but the exact interrelationships of CAD, COPD, and apoM remain elusive and require further investigation. Nevertheless, a previous study showed that *apoM* gene expression is inversely correlated with the severity of COPD [16].

Previously, apoM was considered a negative acute response protein [21]. *ApoM* gene expression is decreased in systemic inflammation stimulated by lipopolysaccharides (LPS), zymosan, or turpentine, as well as in patients with sepsis [21, 22]. In this study, the apoM level was correlated with the amount of hs-CRP, which is a marker of systemic inflammation, but not with that of pentraxin-3, a marker of acute-phase immune reactions [23]. Many reasons may explain this discrepancy. The previous studies showing decreased *apoM* gene expression after exposure to chemicals or LPS were performed in an acute setting, using compounds known to elicit a massive systemic inflammatory response [21, 22]. In this study, the increased inflammatory state was the result of a chronic state that might have different mechanisms compared to the acute state [24]. In addition, two different protective mechanisms involving apoM take place under acute or chronic inflammatory conditions. In response to acute inflammation, circulating apoM is severely depleted upon binding to S1P and the endothelium-protective S1P1 receptor; these interactions prevent an increase in vascular permeability or leakiness [8, 25]. On the other hand, in the process of chronic systemic inflammation, apoM expression might be compensated or even up-regulated to counteract inflammatory effects over a longer period of time as part of an innate protective mechanism [26]. A previous study showed that elevated *apoM* gene expression is associated with lower FEV_1_/FVC [16], corroborating our findings. Nevertheless, the present study was not designed to determine the the mechanisms that cause apoM elevation in COPD patients, and additional investigations are needed for this purpose. A previous study revealed elevated pentraxin-3 in patients with obstructive sleep apnea and increased carotid intima-media thickness [23], in agreement with the present study; however, additional studies are warranted to characterize the inflammatory response in COPD patients and its relationship with atherosclerosis.

In this study, surprisingly, serum apoM was only associated with one lipid, LDL-C. Serum apoM reportedly shows positive correlations with serum total cholesterol, LDL-C, and HDL-C [7, 11]. This is particularly true for HDL-C, as approximately 96 % of apoM is bound to HDL particles [27]. In COPD patients, it is possible that apoM is readily redistributed within the pool of serum lipoproteins, and this occurs even though it is anchored to the surface of lipoproteins by its hydrophobic signal peptide [28]. ApoM was shown to be exchanged from HDL-C to LDL-C because of altered HDL-C amounts under inflammatory conditions [29, 30]. Accordingly, in this study, HDL-C was lower in COPD patients. On the other hand, LDL-C particles are susceptible to oxidative modifications [31] and a decrease in particle size [32] during infection and inflammation, all of which increase the likelihood of their participation in the development of atherosclerosis [33]. Indeed, small-sized LDL-C particles (known as subclass pattern B) more effectively cross the endothelial barrier [34] and are more susceptible to oxidative modifications [35]. This could result in a rapid uptake and accumulation of cholesterol in macrophages and, consequently, contribute to the development of atherosclerosis [36], which should be examined in future studies.

Finally, the abnormal lipid profile observed in this study suggests a high risk for cardiovascular diseases in COPD patients. Indeed, HDL-C is inversely associated with cardiovascular disease diagnosis [37, 38]; this is attributed primarily to the role of HDL-C in the reverse transport of cholesterol [39]. Increased Lp(a) is another strong independent risk factor for CAD [40]. The interaction of Lp(a) with pro-inflammatory oxidized phospholipids is thought to be atherogenic [41]. The decreased HDL-C levels and increased Lp(a) levels observed in the current study suggest an increased risk of cardiovascular in COPD patients. Systemic inflammation is strongly associated with atherosclerosis [36] and might explain the high risk of cardiovascular diseases associated with the pathological process of COPD.

A few limitations of the present study should be mentioned. The main limitation is the disproportionate number of men included in the study population. Women only accounted for 14 % of all subjects. A study design with an approximately equal number of male and female subjects would provide more conclusive results. Another limitation is the relatively small number of COPD patients with CAD. Additional studies with a larger sample size for this COPD subpopulation will be necessary to elucidate the possible role of apoM in cardiac function. In this study, apoM was not associated with CAD when considering the use of drugs in the multivariate model. Longitudinal studies could be particularly helpful in determining the causal relationships of apoM, COPD, and CAD.

## Conclusions

Serum apoM was elevated in COPD patients, and it increased gradually with the stage of COPD severity. It was also found to be an independent risk factor for COPD development. There was no association between apoM and the development of CAD in COPD patients.

### Ethics approval and consent to participate

This study protocol was reviewed and approved by The Second Xiangya Hospital Investigational Review Board. Written informed consent was obtained from all participants.

### Consent for publication

Not applicable.

### Availability of data and materials

The dataset supporting the conclusions of this article is included within the article and its additional file.

## Additional file

Additional file 1: Table S1. Clinical characteristics of COPD patients by GOLD stage. (DOC 38 kb)

## Abbreviations

ANOVA: analysis of variance
apoB: apolipoprotein B
apoM: apolipoprotein M
CAD: coronary artery disease
COPD: chronic obstructive pulmonary disease
CRP: C-reactive protein
FEV_1_: forced expiratory volume in one second
FVC: forced vital capacity
GOLD: Global Initiative for Chronic Obstructive Lung Disease
HDL: high-density lipoprotein
HDL-C: high-density lipoprotein cholesterol
hs-CRP: high-sensitivity C-reactive protein
LDL-C: low-density lipoprotein cholesterol
NS: non-significant
OD: optical density
