# Supplementary material for: High apolipoprotein M serum levels correlate with chronic obstructive pulmonary disease
Source: Lipids Health Dis. 2016 Mar 22;15:59. doi: 10.1186/s12944-016-0228-1 (PMC4802916; doi:10.1186/s12944-016-0228-1)
Supplement: Additional file 1: Table S1. — Clinical characteristics of COPD patients by GOLD stage. (DOC 38 kb) [file 12944_2016_228_MOESM1_ESM.doc]

Additional file 1: Table S1. Clinical characteristics of COPD patients by GOLD stage

|  | GOLD IV | GOLD III | GOLD I/II |
| --- | --- | --- | --- |
| **Subjects, n** | 39 | 39 | 32 |
| **Demographics** |  |  |  |
| Age, years | 66±10 | 69 ± 9 | 68±10 |
| Sex, % male | 90 | 90 | 78 |
| Body mass index, kg/m^2^ | 21.6±2.5 | 22.4 ± 2.5 | 22.6 ± 1.9 |
| Smoking history, % | 59 | 74 | 53 |
| **Comorbidities, %** |  |  |  |
| CAD | 23 | 38 | 22 |
| Hypertension | 21 | 38 | 19 |
| Diabetes mellitus | 3 | 8 | 3 |
| Hormone therapy | 38 | 23 | 19 |

GOLD, Global Initiative for Chronic Obstructive Lung Disease; COPD, chronic obstructive pulmonary disease; CAD, coronary artery disease.

Data are mean±standard deviation for continuous variables and percentage for categorical variables.
